# Using persistent photoconductivity to write a low-resistance path in SrTiO_3_

**DOI:** 10.1038/s41598-017-07090-2

**Published:** 2017-07-27

**Authors:** Violet M. Poole, Slade J. Jokela, Matthew D. McCluskey

**Affiliations:** 1 Department of Physics and Astronomy, Washington State University, Pullman, WA 99164-2814 USA; 2 Klar Scientific LLC, 1615 NE Eastgate Blvd., Pullman, WA 99163 USA

## Abstract

Materials with persistent photoconductivity (PPC) experience an increase in conductivity upon exposure to light that persists after the light is turned off. Although researchers have shown that this phenomenon could be exploited for novel memory storage devices, low temperatures (below 180 K) were required. In the present work, two-point resistance measurements were performed on annealed strontium titanate (SrTiO_3_, or STO) single crystals at room temperature. After illumination with sub-gap light, the resistance decreased by three orders of magnitude. This markedly enhanced conductivity persisted for several days in the dark. Results from IR spectroscopy, electrical measurements, and exposure to a 405 nm laser suggest that contact resistance plays an important role. The laser was then used as an “optical pen” to write a low-resistance path between two contacts, demonstrating the feasibility of optically defined, transparent electronics.

## Introduction

STO is a complex oxide semiconductor with the perovskite structure and an indirect band gap of 3.2 eV^1, 2^. STO has been used as a photo-catalyst to hydrolyze water^3^, a material for high-temperature oxygen sensors^4^, and a substrate for oxide thin films^5^. The electrical properties are tunable from an insulator to metallic-like conduction^6, 7^, and the interface between SrTiO_3_ and insulating oxides can form a highly conductive layer^8–10^. Cr-doped STO has been shown to have bi-stable resistance that could potentially be used for resistance change memory^11^.

Annealing STO at 1200 °C produces defects that give rise to persistent photoconductivity (PPC), with sub-gap light exposure causing the free-electron concentration to increase by a factor of several hundred^12^. The free carriers persist for days or weeks after the light is turned off, at room temperature. The specific defect that causes PPC has not been identified, but the results are consistent with a native acceptor defect such as a *V*_Ti_–*V*_O_ pair^13^. Annealing in vacuum prior to the 1200 °C anneal yields optimal PPC behavior^14^. The vacuum anneal introduces oxygen vacancies^15^, which are necessary for PPC, consistent with the *V*_Ti_–*V*_O_ model.

Prior work on large PPC has focused on *DX* centers, deep-level defects that involve the displacement of a donor impurity^16, 17^. Their metastable behavior stimulated interest in using *DX* centers for holographic data storage, where bits are optically written and read within the volume of a crystal^18, 19^. Because *DX* centers exhibit PPC, optical exposure increases the free-carrier density and thus changes the refractive index^20^. The change in refractive index is then read out optically. In addition to holographic memory, 3D electronic architectures could be defined optically, with current pathways traveling throughout the bulk of a crystal. The main practical problem, however, is that the *DX* barrier is too small (~0.2 eV). At room temperature, the barrier is surmounted quickly, so the sample does not show PPC.

**Figure 1:**
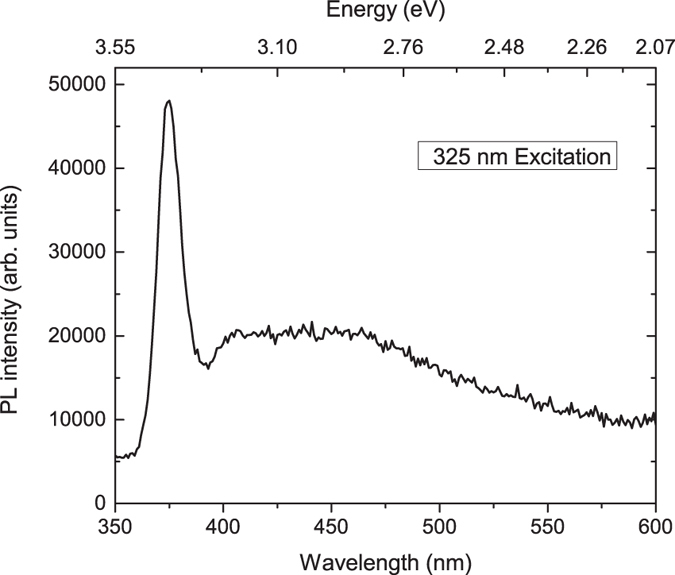
Typical PL spectrum of STO, after annealing and light exposure.

In the present work, *room temperature PPC* was utilized to selectively reduce two-point resistance values. Because secondary luminescence from the sample might affect this process, we performed PL spectroscopy to characterize the samples. A typical PL spectrum is displayed in Fig. 1, which shows a peak at 3.2 eV and a broad band at 2.9 eV. These features have been attributed to the radiative recombination of an exciton bound to a defect such as an oxygen vacancy^21, 22^. The 2.9 eV peak, in particular, is relevant to this study because its photon energy can induce PPC.

**Figure 2:**
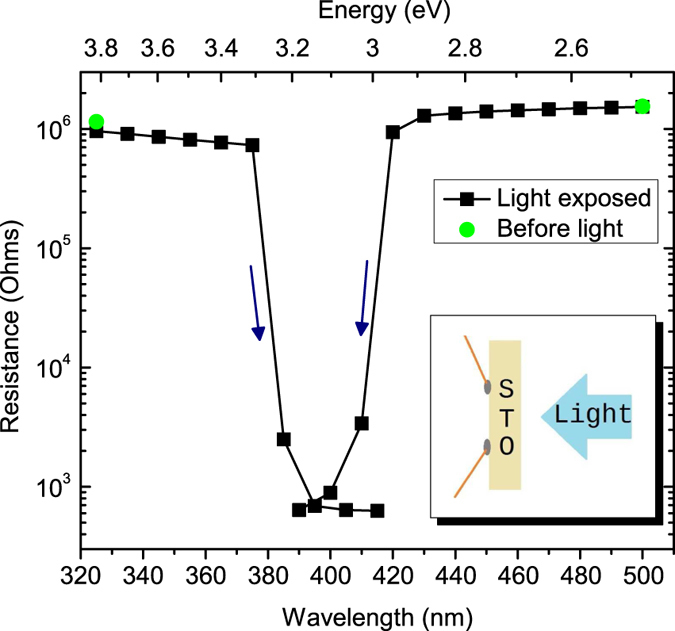
Two-point resistance versus wavelength of light for two STO samples. The duration of light exposure was 10 s at each wavelength. Arrows indicate whether wavelengths were increased or decreased sequentially. Inset: Schematic diagram of the setup. Contacts were placed on the side opposite to light exposure.

Figure 2 shows the effect of light exposure on resistance. Light impinged on the side opposite to the contacts (Fig. 2 inset). This geometry prevents the complication of shadowing by the contacts. Light exposure started with a wavelength of 325 nm for 10 s, and proceeded by successively increasing the wavelength in steps of 10 nm. Significant resistance change did not occur until 385 nm (3.22 eV), which is near the band gap. The penetration depth at 325 nm is tens of nm, while at 355 nm it is ~1 μm^2^. Therefore, the crystal acts as a long-pass filter and blocks above-gap photons. Since the light is absorbed well before it reaches the region near the contacts, the measured resistance remains high. The PL (Fig. 1) does appear to induce some PPC. As shown in Fig. 2, however, this secondary effect causes only a minor drop in resistance.

Figure 2 also shows the light exposure threshold starting from the low-energy side. Exposure started at a wavelength of 500 nm for 10 s, decreasing by steps of 10 nm. Only small changes were observed in the resistance values until 420 nm. With 410 nm light, the change is very significant (3 orders of magnitude). This threshold for PPC, 2.9–3.0 eV, is below the band gap (3.2 eV) and consistent with the value obtained by IR absorption^12^.

**Figure 3:**
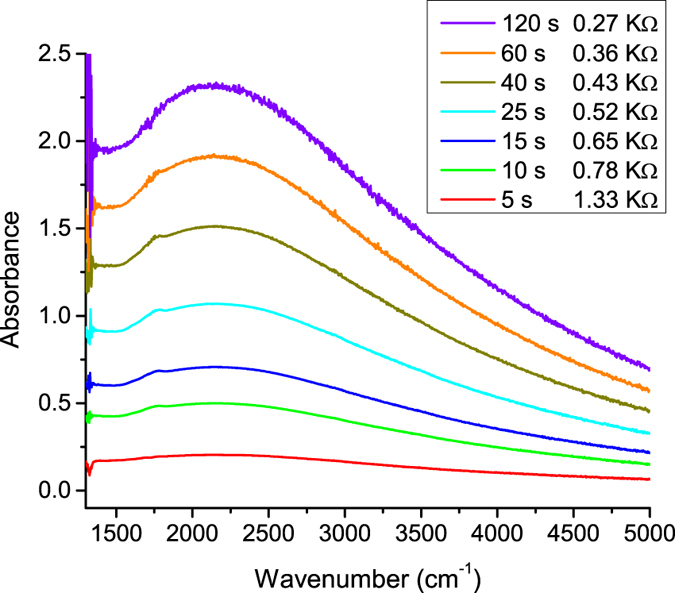
IR absorption and two-point resistance values as a function of light exposure duration (405 nm wavelength). The crystal had a resistance of 2.56 MΩ prior to light exposure. An IR spectrum from the unexposed crystal was used as a reference for the absorbance plots. Note: 1000 cm^−1^ = 0.124 eV.

The strength of mid-IR absorption is proportional to the carrier density^23^. Successively measuring the IR spectrum after incremental light exposure allowed us to correlate the change in electrical resistance with the free-electron density. First, a reference IR spectrum was obtained prior to light exposure. This sample had a resistance of 2.46 MΩ. A 405 nm light-emitting diode was then turned on for several seconds and another spectrum was taken. The first 5 s of light exposure dropped the resistance from 2.46 MΩ to 1.33 KΩ, while only a small change in free-carrier absorption was observed (Fig. 3). This procedure was repeated until the absorbance reached a saturation value.

**Figure 4:**
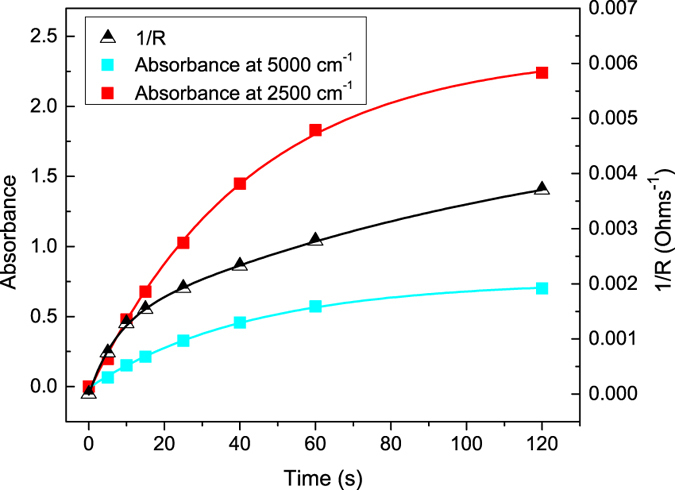
IR absorption (left axis) and conductance (right axis) versus light exposure duration (405 nm wavelength). Solid lines are exponential fits to the data.

Plots of absorbance versus exposure time are shown for two different wavenumber values, 2500 and 5000 cm^−1^ (Fig. 4). They both have the same single-exponential time constant of 43 s, consistent with excitation from a single defect level to the conduction band. Figure 4 also shows a plot of conductance (1/*R*) versus exposure time. These data were fit with two exponential functions, with time constants of 7 and 122 s. While the free-carrier absorption and conductance both show monotonically increasing behavior, they do not correlate exactly. Specifically, the contact resistance has a rapid initial decrease (short time constant). This observation suggests that the contact resistance has a nonlinear dependence on free-carrier concentration or that interface traps are filled during the first few seconds of light exposure.

**Figure 5:**
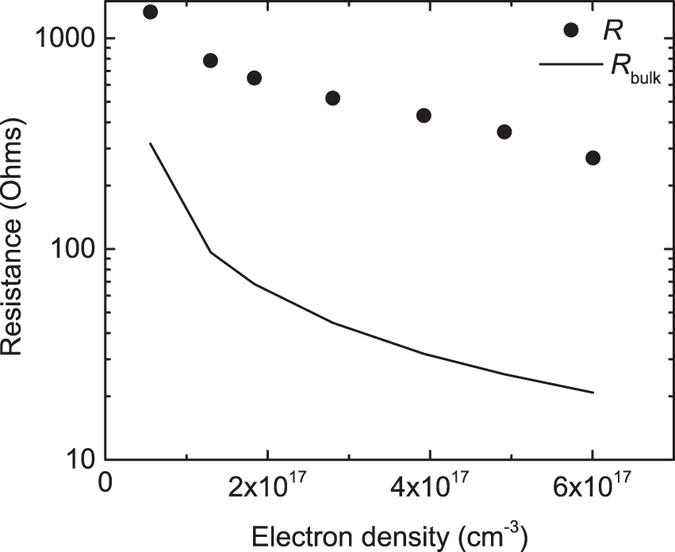
Measured resistance (*R*) versus photogenerated electron density. The electron density was determined from the IR absorbance (ref. 24). The solid line is an estimate of the bulk resistance (*R*_bulk_) from Eq. (2). The higher values of *R* suggest that the contact resistance is significant.

To further explore this effect, we plotted resistance *R* versus carrier density *n*, where *n* was determined from the IR absorbance^24^. The resistivity is given by

1 $${\rm{\rho }}=\frac{1}{ne{\rm{\mu }}},$$

where μ = 5 cm^2^/Vs is the mobility at room temperature^25^. In the absence of contact resistance, the measured two-point resistance can be estimated as (Supplementary Information)

2 $${R}_{{\rm{bulk}}}=\frac{{\rm{\rho }}}{{\rm{\pi }}{\rm{\delta }}}\,\mathrm{ln}(\frac{d-a}{a}),$$

where *d* is the distance between the contacts, *a* is the contact radius, and δ is the sample thickness. Using *d* = 5 mm, *a* = 0.5 mm, and δ = 0.5 mm, Eq. (2) yields *R*_bulk_ ≈ (14 cm^−1^)ρ. This estimate is significantly below the measured resistance (Fig. 5), suggesting that contact resistance is important. The contact resistance may involve a defective, resistive layer at the STO surface^26, 27^, perhaps introduced during the thermal treatments. Regardless of the mechanism, it is clear that the contact resistance shows the same overall trend as the bulk; namely, a monotonic, persistent drop versus light exposure.

**Figure 6:**
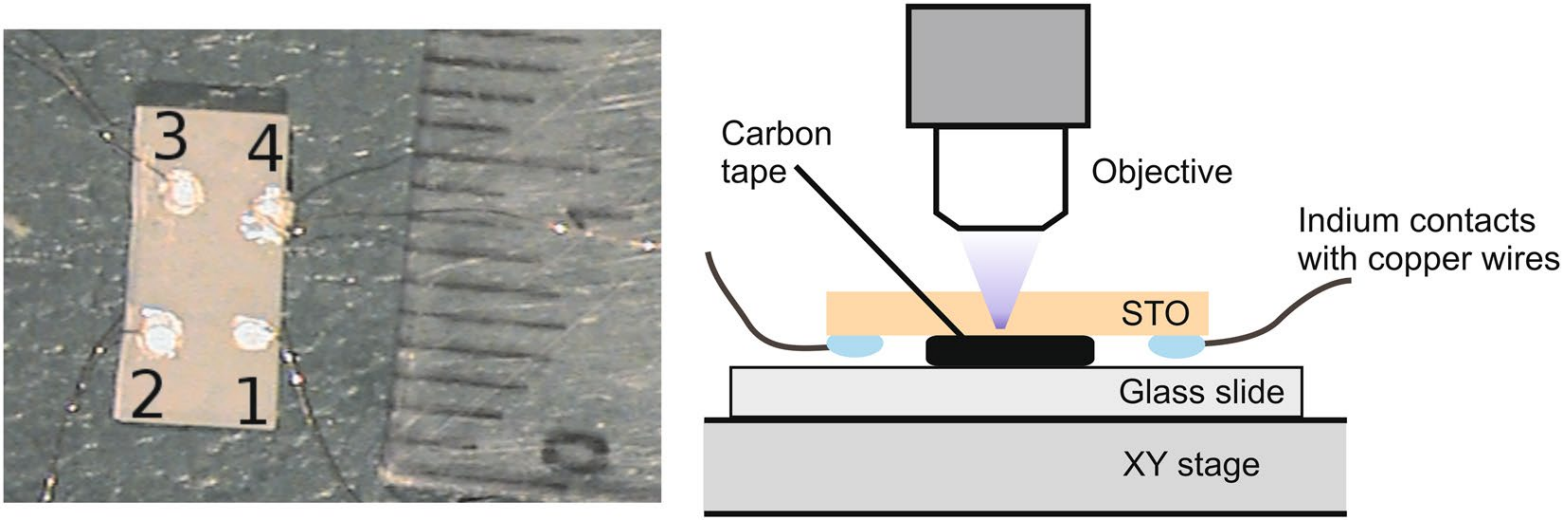
Left: Photograph of STO crystal with contacts used for the photo-lithography experiment. Contacts are labeled 1–4. A ruler is next to the crystal for scale; each black line is 1 mm. Right: Schematic diagram of the experiment.

To demonstrate the possibility of lithography, PPC was induced by focusing a 405 nm laser on specific regions of a sample. We first exposed contact 2 (from the back) for 3 s, turned off the laser, and measured the resistance (Fig. 6). Then contact 1 was exposed for 3 s and resistance was measured again. Finally, a path was traced from contact 1 to contact 2. To test the persistence, measurements were made 1 and 5 days (in the dark) after the exposures. Values are given in Table 1.

**Table 1:** Resistance values after exposure with a 405 nm laser diode (see Fig. 6).

Exposure Step	Resistance between 1 and 2	Resistance between 3 and 4
Before light exposure	1.01 MΩ	1.77 MΩ
Expose contact # 2	0.61 MΩ	1.73 MΩ
Expose contact # 1	2.31 KΩ	1.72 MΩ
Draw path from 1 to 2	0.59 KΩ	1.40 MΩ
1 Day Later	0.97 KΩ	1.62 MΩ
5 Days Later	1.40 KΩ	1.72 MΩ

By successively exposing each contact, then writing a path, one can see that the two-point resistance is affected by contact and bulk resistance. Exposing the contacts resulted in a 400-fold decrease in resistance, although light scattering probably reduced the resistivity of the material between the contacts as well. Drawing a path between the contacts further reduced the resistance by a factor of 4. The resistance between contacts 3 and 4 changed very little when the path was created between contacts 1 and 2, verifying electrical isolation. The 3 order-of-magnitude change in resistance persists for 1 day and is still significant after 5 days.

In conclusion, we have demonstrated that PPC can be exploited to write a high-conductivity pathway using an optical pen. The defined path persists for several days at room temperature and does not affect the transparency of the crystal. In principle, this technique could be used to create transparent electronic circuits that could be erased (e.g., by resistive heating) and re-written.

## Methods

All measurements were performed at room temperature. (100) oriented STO crystals with one side finely polished were obtained from MTI. Samples were first placed in an evacuated ampoule (50–100 mTorr) and vacuum annealed at 800 °C for 1 hr. Then samples were placed in an evacuated ampoule with 0.6 g SrO powder and annealed at 1200 °C for 1 hr. After the two-step annealing process, crystals were cut into two pieces. Contacts of pressed indium were applied to the rough side of the annealed sample and copper wires were attached with silver paint. These wires were attached to a Keithley model 2400 source meter, which is capable of measuring resistance up to 200 MΩ. Current-voltage plots taken before and after light exposure confirmed that the pressed indium contacts showed nearly ohmic behavior (Supplementary Information). Prior studies of pressed indium contacts on heavily Nb-doped STO also showed nearly ohmic behavior but with lower contact resistance values^28^.

For the monochromatic light source and PL measurements, we used a JY-Horiba FluoroLog-3 spectrofluorometer system equipped with double grating excitation and emission monochromators. Different pieces of the same crystal were used for the IR experiment and the photo-lithography experiment. To measure IR spectra and resistance simultaneously, a 405 nm high brightness light emitting diode (LED) was placed approximately 2.5 cm from the polished side of the sample inside the compartment of a Bomen DA8 vacuum Fourier transform spectrometer (FTIR). When on, the LED was powered with approximately 20 mA and 3.4 V. The exposure geometry was the same as that shown in Fig. 2 (inset).

For the photo-lithographic experiment, 4 indium contacts were pressed on the rough side of the annealed STO crystal, and wires were attached with silver paint. The crystal was attached to a glass microscope slide with the contacts facing downwards with double-sided carbon sticky tape. The tape was insulating and did not affect the electrical measurements. The slide was placed in a PL microscope (Klar Scientific, 10x objective, numerical aperture 0.2) with an XY stage and a 405 nm laser diode (5 mW) as the excitation source. The laser spot at the back of the contact had a diameter of ~5 μm and was surrounded by a weaker spot (~1 mm diameter) of scattered light.

### Data Availability Statement

The datasets generated during and/or analysed during the current study are available from the corresponding author on reasonable request.

## Electronic supplementary material

Current-voltage information
