# Supplementary material for: Using persistent photoconductivity to write a low-resistance path in SrTiO3
Source: Sci Rep. 2017 Jul 27;7:6659. doi: 10.1038/s41598-017-07090-2 (PMC5532236; doi:10.1038/s41598-017-07090-2)
Supplement: Supplementary file 1 — Current-voltage information [file 41598_2017_7090_MOESM1_ESM.pdf]

Using persistent photoconductivity to write a low-resistance path in $SrTiO_3$

Violet M. Poole,[1] Slade J. Jokela,[2] and Matthew D. McCluskey[1,*]

[1]*Department of Physics and Astronomy, Washington State University, Pullman, WA 99164-2814*

[2]*Klar Scientific LLC, 1615 NE Eastgate Blvd., Pullman, WA 99163*

*mattmcc@wsu.edu

The measured two-point resistance is given by[1]

$$R = V/I = 2R_C + R_{\mathrm{bulk}} \tag{1}$$

where R_C is the resistance of one contact, R_{bulk} is the resistance of the sample, and the resistance of the wires is neglected. As shown in Figure 1, the *I-V* relations for our samples show nearly linear behavior. This implies that the contacts are "ohmic" as opposed to Schottky barriers.

Consider a sample of thickness δ with a circular contact of radius *a*. Current flows through the contact and spreads out radially (Figure 2). Assuming a thin sample ($s >> \delta$), the voltage drop is

$$V_1 = \int IdR = \int_a^s I\rho\left(\frac{dr}{2\pi r\delta}\right) = \frac{I\rho}{2\pi\delta}\ln\left(\frac{s}{a}\right) \tag{2}$$

For two contacts, the voltage drop is twice that given in Equation (2),

$$V = \frac{I\rho}{\pi\delta}\ln\left(\frac{s}{a}\right) \tag{3}$$

Assuming zero contact resistance, Equations (1) and (3) yield

$$R_{\text{bulk}} = \frac{\rho}{\pi\delta}\ln\left(\frac{s}{a}\right) \tag{4}$$

Letting *d* equal the distance between the centers of the contacts,

$$R_{\text{bulk}} = \frac{\rho}{\pi\delta}\ln\left(\frac{d-a}{a}\right) \tag{5}$$

Note that the logarithm is insensitive to the exact values of *d* and *a*.

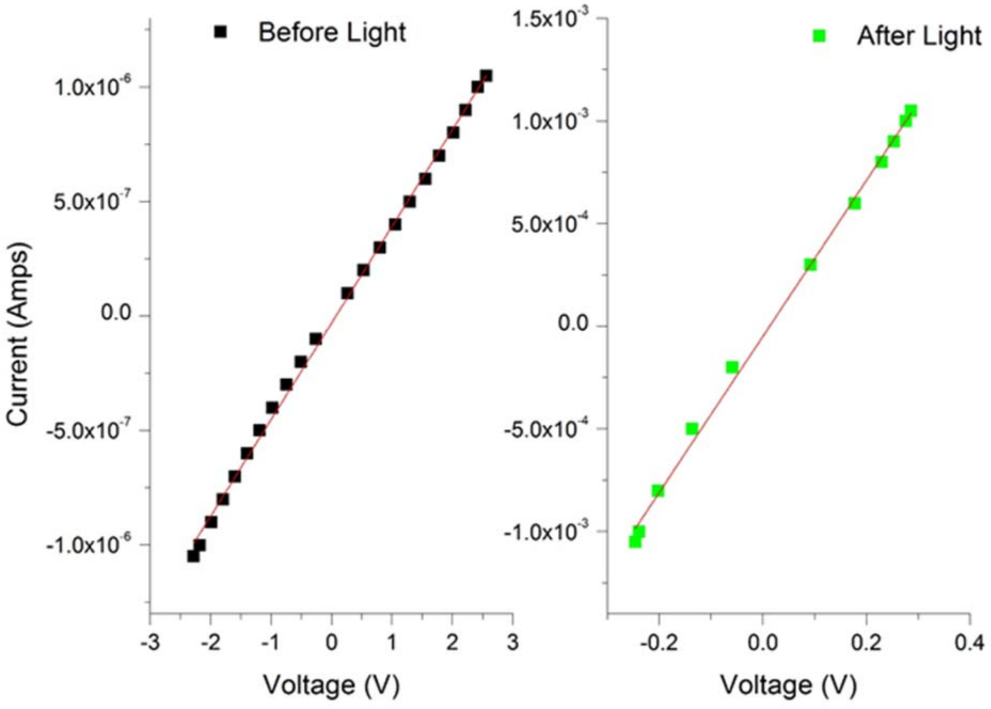

Figure 1. Typical current-voltage curves of STO before and after light exposure, showing nearly ohmic (linear) behavior.

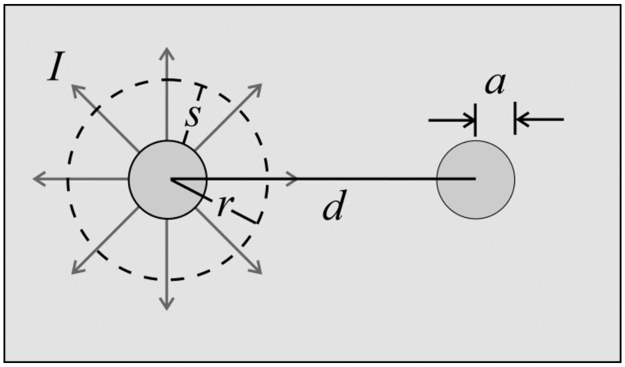

Figure 2. Schematic diagram of a two-point resistance measurement (top view). Current is shown flowing from the left contact. Current also flows into the right contact (not shown).

[1] D.K. Schroder, *Semiconductor Material and Device Characterization*, 3rd edition (John Wiley & Sons, 2006), pp. 2-8.
